# Leaf Morphology, Photosynthetic Performance, Chlorophyll Fluorescence, Stomatal Development of Lettuce (*Lactuca sativa* L.) Exposed to Different Ratios of Red Light to Blue Light

**DOI:** 10.3389/fpls.2016.00250

**Published:** 2016-03-10

**Authors:** Jun Wang, Wei Lu, Yuxin Tong, Qichang Yang

**Affiliations:** ^1^Institute of Environment and Sustainable Development in Agriculture, Chinese Academy of Agricultural SciencesBeijing, China; ^2^Key Laboratory of Energy Conservation and Waste Management of Agricultural Structures, Ministry of AgricultureBeijing, China

**Keywords:** R/B ratio, photosynthetic performance, chlorophyll fluorescence, stomata, dry weight, *Lactuca sativa* L.

## Abstract

Red and blue light are both vital factors for plant growth and development. We examined how different ratios of red light to blue light (R/B) provided by light-emitting diodes affected photosynthetic performance by investigating parameters related to photosynthesis, including leaf morphology, photosynthetic rate, chlorophyll fluorescence, stomatal development, light response curve, and nitrogen content. In this study, lettuce plants (*Lactuca sativa* L.) were exposed to 200 μmol⋅m^−2^⋅s^−1^ irradiance for a 16 h⋅d^−1^ photoperiod under the following six treatments: monochromatic red light (R), monochromatic blue light (B) and the mixture of R and B with different R/B ratios of 12, 8, 4, and 1. Leaf photosynthetic capacity (*A*_max_) and photosynthetic rate (*P*_n_) increased with decreasing R/B ratio until 1, associated with increased stomatal conductance, along with significant increase in stomatal density and slight decrease in stomatal size. *P*_n_ and *A*_max_ under B treatment had 7.6 and 11.8% reduction in comparison with those under R/B = 1 treatment, respectively. The effective quantum yield of PSII and the efficiency of excitation captured by open PSII center were also significantly lower under B treatment than those under the other treatments. However, shoot dry weight increased with increasing R/B ratio with the greatest value under R/B = 12 treatment. The increase of shoot dry weight was mainly caused by increasing leaf area and leaf number, but no significant difference was observed between R and R/B = 12 treatments. Based on the above results, we conclude that quantitative B could promote photosynthetic performance or growth by stimulating morphological and physiological responses, yet there was no positive correlation between *P*_n_ and shoot dry weight accumulation.

## Introduction

As a signal and energy source, light is one of the most important environment factors for plant growth and development. Compared with light intensity and photoperiod, light quality shows much more complex effects on plant morphology and physiology. Specific spectrum stimulates different morphological and physiological responses. Red light (R) and blue light (B) absorbed by photosynthetic pigments are more effective than other wavelengths (Pfündel and Baake, 1990). It is well known that R influences stem elongation, root to shoot ratio, chlorophyll content, photosynthetic apparatus (Appelgren, 1991; Aksenova et al., 1994; Sæbø et al., 1995). B causes physiological responses via phototropins, including phototropism, hypocotyl elongation, leaf expansion, stomatal opening, leaf anatomy, enzyme synthesis, chloroplast movements, and genes expression (Christie, 2007; Inoue et al., 2008; Wang et al., 2009).

However, monochromatic R or B could not meet the requirement of plant growth. Plants under R alone displayed abnormal leaf morphology and reduced photosynthetic rate (*P*_n_) compared with white light or R supplemented with B (Goins et al., 1998; Wang et al., 2009, 2015; Hogewoning et al., 2010b). Hogewoning et al. (2010b) reported that leaf photosynthetic machinery dysfunction appeared under R alone, only 7% B was sufficient to prevent any overt dysfunctional photosynthesis. In addition, B alone could also reduce *P*_n_ in many species, such as chrysanthemum plantlets (Kim et al., 2004), *Withania Somnifera* (L.) plantlets (Lee et al., 2007). B involves inhibition of cell expansion or division (Appelgren, 1991; Folta et al., 2003; Dougher and Bugbee, 2004), therefore a reduction in B could increase leaf area (LA; Dougher and Bugbee, 2001; Hogewoning et al., 2010a; Hernández and Kubota, 2014), which promotes light interception and dry weight accumulation.

It has been reported that *P_n_* and shoot dry weight could be increased by mixture of R and B compared with monochromatic light (Brown et al., 1995; Goins et al., 1997; Ohashi-Kaneko et al., 2006; Hogewoning et al., 2010b; Nanya et al., 2012; Li et al., 2013). However, there are discrepancies for different plants in response to B dose in the background R. It has been reported that the optimal R/B ratio for fresh and dry weight accumulation in strawberry plantlet, rapeseed (*Brassica napus* L.) plantlets *in vitro*, and cucumber seedlings was 7/3 (Nhut et al., 2003), 1/3 (Li et al., 2013), and 9 (Hernández and Kubota, 2016), respectively.

Lettuce as a fresh salad food is an important vegetable throughout the world because of its fast growth and commercial value, and it is known to be sensitive to light quality as a model crop (Dougher and Bugbee, 2001; Lin et al., 2013). For lettuce, addition of B under R could inhibit hypocotyl extension and cotyledon elongation (Hoenecke et al., 1992), increase dry weight, LA, and leaf number (Yorio et al., 1998, 2001; Johkan et al., 2010; Wojciechowska et al., 2015). In contrast, it also been reported that greater dry weight and LA under R treatment were observed than those under mixture of R and B treatments (Ohashi-Kaneko et al., 2007; Son and Oh, 2013; Wollaeger and Runkle, 2014). Therefore, the optimal R/B ratio under a combination of red and blue light-emitting diodes (LEDs) is not yet determined. And few studies have been reported about the effects of different R/B ratios on leaf photosynthetic performance of lettuce.

The objective of the present study was to determine the effects of different R/B ratios on morphology and photosynthetic performance of lettuce by investigating photosynthetic rate, chlorophyll fluorescence, stomatal development, light response curve, and nitrogen content. The results of this study would be used to give guidance on light sources design for lettuce cultivation in a controlled environment.

## Materials and Methods

### Plant Material and Experimental Setup

Lettuce (*Lactuca sativa* L.) seeds were sown in substrate containing a mixture of vermiculite and peat (3:1, V/V), and geminated under 150 μmol⋅m^−2^⋅s^−1^ irradiance provided by fluorescent lamps (TL-D 36W, Philips) in a controlled environment. After the second leaf was fully expanded, the seedlings were randomly divided into six groups and transferred to six separate hydroponic (Yamasaki lettuce nutrient solution; pH≈5.8; EC≈1.5 mS^.^cm^−1^) systems in a controlled environment. Air temperature was 24°C during photoperiod and 20°C during dark period. Photoperiod, relative humidity and CO_2_ concentration were 16 h⋅d^−1^, 60%, and 400 μmol⋅mol^−1^, respectively. LEDs were equipped with light plates (Dongguan Bio-lighting Sciences and Technology Co. Ltd, China) and power DC supply (PKU-MS605D). Irradiance of R and B was individually controlled by adjusting electric current of power DC supply for each treatment. LEDs provided R with peak wavelength of 657 nm and B with peak wavelength of 450 nm. All plants were subjected to 200 μmol⋅m^−2^⋅s^−1^ irradiance measured by spectrometer (AvaSpec-2048-USB2, the Netherland) at the top of the canopy. Six light quality treatments based on different R/B ratios, were created and labeled as R, R/B = 12, R/B = 8, R/B = 4, R/B = 1, and B. Lettuce plants were grown for 30 days after transplanting before harvest. All the treatments were repeated twice.

### Chlorophyll Concentration

Samples were excised from the leaves of 10 plants at a similar position for each treatment. Leaves were weighed out in 0.1–0.2 g (fresh weight, FW). The extractions were performed using 10 ml (V) of 80% acetone until the leaf turned white. The optical density was measured with UV-1800 spectrophotometer (Shimadzu, Japan) at 663nm (OD663) and at 645 nm (OD645) for chlorophyll a (Chl a) and chlorophyll b (Chl b). The chlorophyll concentrations (Chl) were determined using (Lichtenthaler and Wellburn, 1983):

(1) $$\begin{array}{cc} Chl\;a\left(mg\cdot g^{-1}\right)=\frac{(12.72\times OD_{663}-2.59\times OD_{645})\cdot V}{1000\times W} & \end{array}$$

(2) $$\begin{array}{cc} Chl\;b\left(mg\cdot g^{-1}\right)=\frac{(22.88\times OD_{645}-4.67\times OD_{663})\cdot V}{1000\times W} & \end{array}$$

Where *V* is the total volume of acetone extract (ml) and *W* is FW (g) of sample.

### Photosynthetic Characteristics and Chlorophyll Fluorescence

Photosynthetic light response curves and photosynthetic characteristics were measured on fully expanded second leaves of four plants from each treatment using the method of Li et al. (2014) with slight modification. The light response curve was measured by using 10 light intensities in the range from 0 to 1200 μmol⋅m^−2^⋅s^−1^. The starting light intensity was 200 μmol⋅m^−2^⋅s^−1^, followed by 100, 50, 25, 0, 400, 600, 800, 1000, and 1200 μmol⋅m^−2^⋅s^−1^. Measurements of photosynthetic light response curves and photosynthetic characteristics were all performed on a single leaf exposed to light source (10% B, 90% R) provided by a portable photosynthesis system (Li-6400, Li-Cor Inc., Lincoln, NE, USA). Leaf temperature and CO_2_ concentration in the leaf chamber were 24°C and 400 μmol⋅mol^−1^, respectively. The VPD in the leaf chamber was maintained at 1.1 kPa. Data were taken when *P*_n_ reached steady state at each light intensity level. Samples from each treatment were measured in the order of R, R/B = 12, R/B = 8, R/B = 4, R/B = 1, and B; hereafter the same measurements were repeated three times. When light response curves were measured, data obtained at light intensity of 200 μmol⋅m^−2^⋅s^−1^ were considered as photosynthetic characteristics.

Chlorophyll fluorescence was also measured on the fully expanded second leaves of four plants from each treatment by a portable photosynthesis system (Li-6400, Li-Cor Inc., Lincoln, NE, USA). Saturating flashes (8000 μmol⋅m^−2^⋅s^−1^) were applied to determine the maximum fluorescence yield during actinic light (*F*_m_′). The effective quantum yield of PSII (ΦPSII) was calculated: ΦPSII = (*F*_m_′-*F*_s_)/*F*_m_′ (Genty et al., 1989). *F*_s_ is the light-adapted steady state fluorescence.

### Stomata Characteristic and Leaf Gas Exchange

Samples were excised from the fully expanded second leaves of four plants at a similar position for each treatment. To observe the stomata, transparent nail polish was smeared on the surface of the leaves. The slides made by the leaf epidermal fingerprint with the transplant nail polish method (Zeng et al., 2008) were observed by optical microscope (Olympus DP71, Olympus Inc., Japan). The length, width and density of stomata were measured with Image-Pro Express software (Olympus Inc., Japan).

Stomatal limitation value (*L*_s_) was calculated as 1-*C*_i_/*C*_a_ (Farquhar and Sharkey, 1982), *C*_a_ was CO_2_ concentration in leaf chamber. Apparent mesophyll conductance (*g*_m_) was calculated as *P*_n_/*C*_i_ (Fischer et al., 1998). *C*_i_ was intercellular CO_2_ concentration.

### LMA, Sucrose, Starch, Carbon, and Nitrogen Content

The leaf mass area (LMA) was calculated using (Hernández and Kubota, 2016):

(3) $$\begin{array}{cc} LMA(g\cdot cm^{-2})=\frac{leaf\;dry\;weight}{leaf\;area} & \end{array}$$

Samples were excised from the leaves of four plants for each treatment before the end of dark period. Total sugar was extracted using the method of Li et al. (2013). The sucrose concentration was determined using the resorcinol method and measured at 480 nm. Extraction of starch was obtained by the method of Takahashi et al. (1995). Starch content was calculated by converting glucose to starch equivalents using a factor of 0.9 (Li et al., 2010). The glucose concentration was determined by using the sulfuric acid anthrone method and measured at 620 nm. Leaf nitrogen content was determined with element analyzer (Isoprime GC5, Italy).

### Statistical Analysis

The fitting parameters of light response curve, including photosynthetic capacity (*A*_max_), dark respiration rate (*R*_d_) and photochemical efficiency at low light (α), were fitted with a non-rectangular hyperbola (Thornley, 1976) using the non-linear fitting procedure ARSIN in SAS (SAS Institute Inc. 9.1, Cary, NC, USA).

All measurements were based on four replicate plants. Statistical analysis was subjected to one-way analysis of using variance ANOVA, and significant differences between the means were tested using Duncan’s multiple range test at 95% confidence.

## Results

### Leaf Photosynthesis and *L*_s_

**FIGURE 1:**
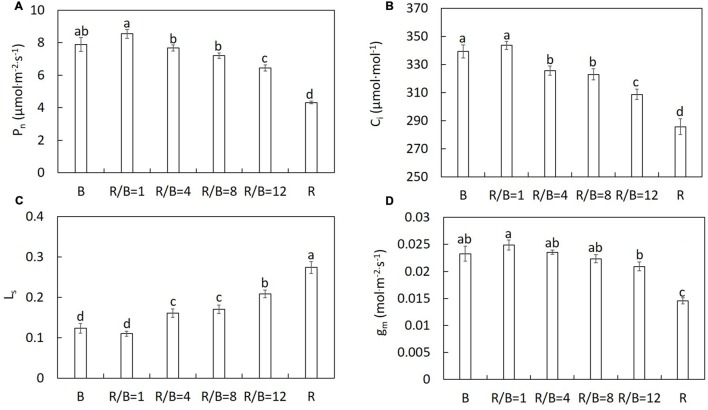
**The effect of different R/B ratios on *P*_n_**(A)**, *C*_i_**(B)**, *L*_s_**(C)**, and *g*_m_**(D)** at the growth irradiance of lettuce.** Values were the means of four replicates with standard errors shown by vertical bars. Different letters indicate significant differences using the Duncan’s Multiple Range Test (*p* < 0.05; *n* = 4).

**FIGURE 2:**
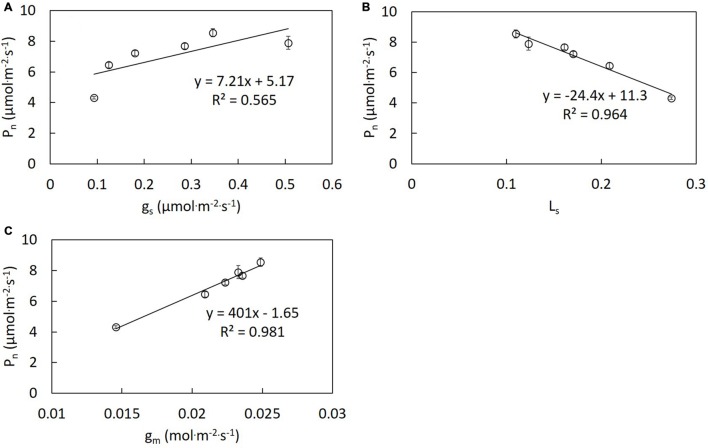
**Correlation analysis between *P*_n_ and *g*_s_ (A), *L*_s_ (B), and *g*_m_ (C) of lettuce grown under different R/B ratios treatments.** Values were the means of four replicates with standard errors shown by vertical bars. Different letters indicate significant differences using the Duncan’s Multiple Range Test (*p* < 0.05; *n* = 4).

The same trend was observed on results of the repeated experiments. Thus, one dataset of the repeated experiments was shown in this study. *P*_n_ differed significantly under different R/B ratios treatments (**Figure 1A**). Decreased R/B ratio led to increasing *P*_n_, except B. Similar trends were observed in *C*_i_, *g*_m_ and stomatal conductance (*g*_s_) in the change of R/B ratio (**Figures 1B,D** and **4**). *P*_n_ under B treatment did not follow the trend of *P*_n_ increasing with decreasing R/B ratio, which was 7.6% lower compared to that under R/B = 1 treatment. *P*_n_ under B and R/B = 1 treatments was 53.2 and 74.0% higher than that under R treatment, respectively. However, *L*_s_ had the opposite trend with decreasing R/B ratio (**Figure 1C**). *P*_n_ correlated positively with *g*_m_ and *g*_s_ and inversely with *L*_s_ (**Figure 2**).

### Growth and Morphology

Growth and morphology in lettuce showed significant difference under different R/B ratios treatments (**Table 1**). Shoot dry weight gradually increased with increasing R/B ratio with the greatest value under R/B = 12 treatment, and no significant difference was observed between R/B = 12 and R treatments. Shoot dry weight under B treatment was 48.1 and 47.2% lower in comparison with those under R/B = 12 and R treatments, respectively. Similar trends were observed for plants in leaf number, LA and LMA. For LMA, there was no significant difference among plants cultured under the six treatments. Chl increased with decreasing R/B ratio. Addition of B increased Chl a/b of lettuce leaves compared to that of R-grown leaves with the lowest value. Chlorophyll content per leaf area (Chl/LA) under mixture of R and B treatments was higher than those grown under monochromatic R or B treatment.

**Table 1:** Effects of different R/B ratios on shoot dry weight, leaf number, Chl, Chl a/b, LA, Chl per leaf area (Chl/LA) and LMA.

R/B ratio	B	R/B = 1	R/B = 4	R/B = 8	R/B = 12	R
Shoot dry weight (g)	0.95^c^	1.04^c^	1.37^b^	1.67^a^	1.83^a^	1.80^a^
Leaf number	20^c^	22^c^	25^b^	30^a^	28^ab^	26^b^
Chl (mg⋅g^−1^ FW)	0.85^a^	0.77^ab^	0.81^a^	0.74^ab^	0.69^bc^	0.60^c^
Chl a/b	3.20^ab^	3.15^ab^	3.28^a^	3.18^ab^	3.00^bc^	2.83^c^
LA (cm^2^)	545^c^	597^c^	771^b^	898^a^	956^a^	950^a^
Chl/LA (g⋅m^−2^)	0.27^bc^	0.30^ab^	0.32^a^	0.32^a^	0.30^ab^	0.24^c^
LMA (g⋅m^−2^)	17.3^a^	17.4^a^	17. 8^a^	18.7^a^	18.8^a^	19.0^a^

*Different letters indicate significant differences using the Duncan’s Multiple Range Test (p < 0.05; n = 4).*

### Photosynthetic Light Response Curves and Fluorescence Characteristics

**FIGURE 3:**
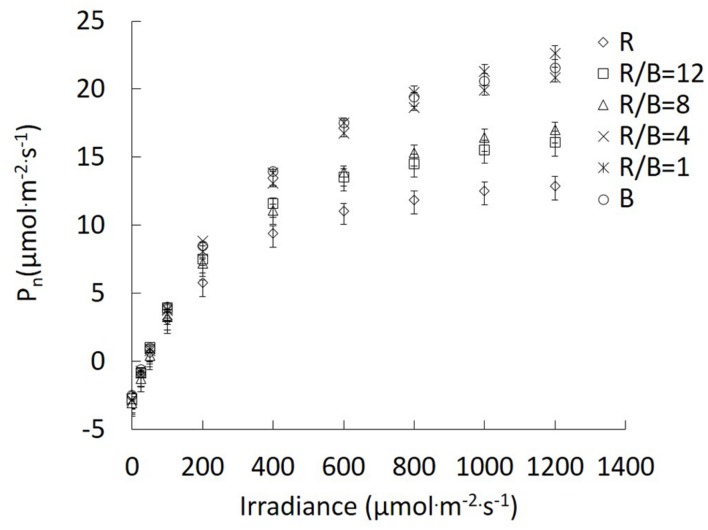
**Response of *P*_n_ to irradiance for lettuce leaves grown under different R/B ratios treatments.** Values were the means of four replicates with standard errors shown by vertical bars (*n* = 4).

Different R/B ratios significantly affected *P*_n_ in the change of irradiance. The differences in *P*_n_ between R and other treatments became increasingly greater with the increase of irradiance. The enhanced effect of decreasing R/B ratio on *P*_n_ was similar with that caused by increasing irradiance (**Figure 3**). For the fitting parameters of photosynthetic light response curve, a reduction in R/B ratio led to increasing *A*_max_, except for B treatment (**Table 2**). *A*_max_ under B and R/B = 1 treatments was 82.7 and 97.8% higher than that under R treatment, respectively. *R*_d_ was the highest under R/B = 8 treatment and the lowest under B treatment. Compared with monochromatic light treatments, α was higher under mixture of R and B treatments, with the maximum value under R/B = 12 treatment.

**Table 2:** Effects of different R/B ratios on the fitting parameters of photosynthetic light response curve, including *A*_max_, *R*_d_ and α.

R/B ratio	B	R/B = 1	R/B = 4	R/B = 8	R/B = 12	R
*A*_max_ (μmol CO_2_⋅m^−2^⋅s^−1^)	27.6^b^	31.3^a^	29.7^ab^	23.8^c^	20.7^d^	18.0^e^
R_d_ (μmol CO_2_⋅m^−2^⋅s^−1^)	2.3^c^	2.7^b^	2.8^b^	3.3^a^	2.7^b^	2.6^bc^
α (μmol photons⋅m^−2^⋅s^−1^)	0.082^b^	0.086^ab^	0.114^ab^	0.108^ab^	0.117^a^	0.084^b^

*Different letters indicate significant difference using the Duncan’s Multiple Range Test (p < 0.05; n = 4).*

Plants grown under B treatment had a lower efficiency of excitation capture by open PSII center (F_v_′/F_m_′) compared to the other treatments. Similar result was observed in ΦPSII (**Table 3**).

**Table 3:** Effects of different R/B ratios on the efficiency of excitation capture by open PSII center (*F*_v_′/*F*_m_′), and ΦPSII.

R/B ratio	B	R/B = 1	R/B = 4	R/B = 8	R/B = 12	R
*F*_v_′/*F*_m_′	0.69^b^	0.76^a^	0.76^a^	0.76^a^	0.77^a^	0.77^a^
ΦPSII	0.62^b^	0.67^a^	0.67^a^	0.67^a^	0.68^a^	0.68^a^

*Different letters indicate significant difference using the Duncan’s Multiple Range Test (p < 0.05; n = 4).*

### Stomatal Characteristics

**FIGURE 4:**
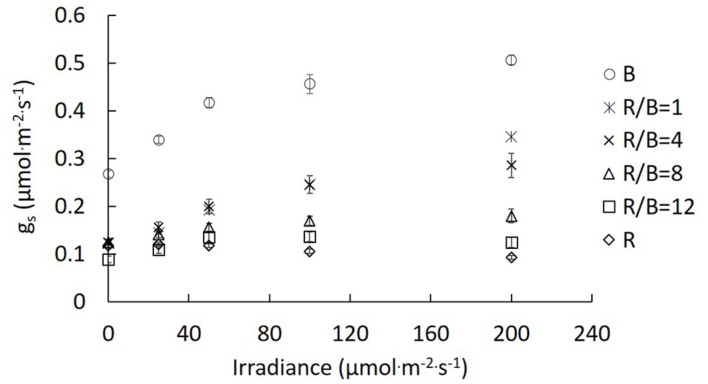
**Response of *g*_s_ to irradiance for lettuce leaves grown under different R/B ratios.** Values were the means of four replicates with standard errors shown by vertical bars (*n* = 4).

*g*_s_ of lettuce leaves had been significantly altered after being exposed to different R/B ratios in the change of irradiance ranging from 0 to 200 μmol⋅m^−2^⋅s^−1^. *g*_s_ increased with increasing irradiance under B, R/B = 1, R/B = 4, R/B = 8, and R/B = 12 treatments, except for R treatment. *g*_s_ under R treatment was almost unresponsive to increasing irradiance. The highest *g*_s_ was observed under B treatments, followed by R/B = 1, R/B = 4, R/B = 8, and R/B = 12, with the lowest value under R treatment at the same measured irradiance (**Figure 4**). The effect of decreasing R/B ratio on *g*_s_ was similar with that caused by increasing irradiance.

As shown in **Table 4**, stomatal development differed greatly under different R/B ratios treatments at the growth irradiance. Decreased R/B ratio gradually caused higher stomatal density on the abaxial and adaxial surfaces. Leaves under B treatment did not follow the trend of stomatal density increasing with decreasing R/B ratio, which were 26 and 42% lower than R/B = 1 treatment on the abaxial and adaxial surfaces, respectively. Stomatal length on the abaxial and adaxial surfaces increased with increasing R/B ratio under mixture of R and B treatments but with no significant differences, which were significantly higher than those under monochromatic R or B treatment. There were no significant differences among stomatal widths on the abaxial and adaxial surfaces of plants cultured under the six treatments. Stomatal pore length and pore width on the abaxial surface under R and B treatments were lower than those under the other treatments. Stomatal pore length and pore width on the adaxial surface increased with increasing R/B ratio under mixture of R and B treatments, but were slightly lower under R treatment. These results indicated that an addition of B under background R could increase stomatal density and stomatal aperture compared with R.

**Table 4:** Effects of different R/B ratios on leaf stomata development. Ab and Ad represented abaxial and adaxial surfaces of lettuce leaves.

R/B ratio	Stomatal length (μm)		Stomatal width (μm)		Pore length (μm)		Pore width (μm)		Stomatal density (stomata mm^−2^)	
	Ab	Ad	Ab	Ad	Ab	Ad	Ab	Ad	Ab	Ad
B	28.0^b^	29.6^b^	20.8^a^	21.2^a^	8.6^ab^	6.4^c^	3.1^b^	2.4^b^	269^b^	253^cd^
R/B = 1	29.4^ab^	29.8^b^	21.8^a^	21.9^a^	9.8^ab^	7.0^bc^	3.8^a^	2.4^b^	363^a^	434^a^
R/B = 4	29.8^ab^	30.9^ab^	21.9^a^	22.0^a^	10.0^ab^	7.1^bc^	4.1^a^	3.1^ab^	283^b^	320^b^
R/B = 8	30.0^ab^	31.2^ab^	22.1^a^	22.1^a^	10.6^a^	8.4^ab^	3.9^a^	3.2^a^	257^bc^	278^bc^
R/B = 12	31.1^a^	32.6^a^	21.9^a^	22.2^a^	10.2^ab^	9.2^a^	3.7^a^	3.0^ab^	223^bc^	230^d^
R	27.9^b^	28.6^b^	20.9^a^	20.6^a^	8.5^b^	7.7^abc^	3.1^b^	2.9^ab^	200^d^	224^d^

*Data were means of 40 fields of view for four samples from each treatment. Different letters indicate significant difference using the Duncan’s Multiple Range Test (p < 0.05; n = 4).*

### NUE, Nitrogen and Carbohydrate Content

**FIGURE 5:**
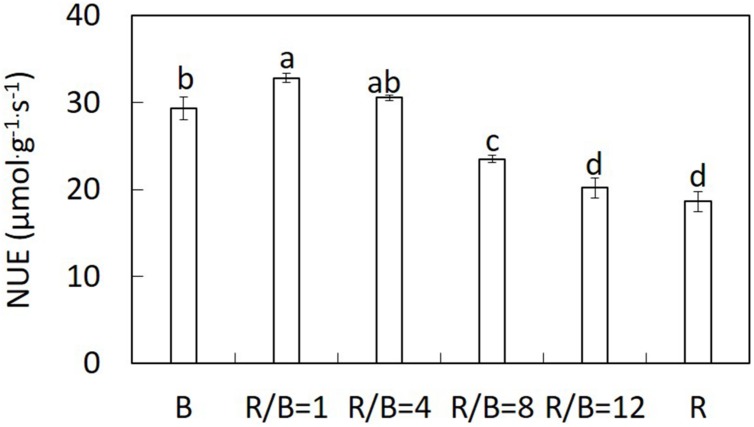
**Effect of different R/B ratios on the ratio of leaf *A*_max_ to nitrogen content per LA (NUE).** Values were the means of four replicates with standard errors shown by vertical bars. Different letters indicate significant difference using the Duncan’s Multiple Range Test (*p* < 0.05; *n* = 4).

The ratio of *A*_max_ to nitrogen content per LA (NUE) increased with decreasing R/B ratio with the highest value under R/B = 1 treatment, but was significantly lower under B treatment (**Figure 5**). NUE under B and R/B = 1 treatments was 57 and 76% higher than that under R treatment, respectively. The accumulation of sucrose under R and R/B = 12 treatments were higher than those under R/B = 8, R/B = 4, R/B = 1, and B treatments. The accumulation of starch was the highest under R treatment and the lowest under B treatment (**Table 5**).

**Table 5:** Effects of different R/B ratios on sucrose and starch content.

R/B ratios	B	R/B = 1	R/B = 4	R/B = 8	R/B = 12	R
Sucrose (mg⋅g^−1^)	0.37^b^	0.34^b^	0.38^b^	0.36^b^	0.53^a^	0.50^a^
Starch (mg⋅g^−1^)	0.10^d^	0.10^d^	0.13^c^	0.16^ab^	0.14^bc^	0.17^a^

*Different letters indicate significant difference by the Duncan’s Multiple Range Test (p < 0.05; n = 4).*

## Discussion

### Additional B had a Greater Impact on *P*_n_ and *A*_max_ by Combination of R

Combination of R and B has been proved to be effective in driving photosynthesis. As shown in **Figure 1A** and **Table 2**, increasing *P*_n_ and *A*_max_ with decreasing R/B ratio until 1 has been observed in the present study. This result indicated that photosynthetic performance of lettuce plant could be efficiently improved by increasing B fraction. The same trends have been reported in rice (Matsuda et al., 2004), and cucumber seedling (Hogewoning et al., 2010b; Hernández and Kubota, 2016). For example, Matsuda et al. (2004) reported that *P*_n_ in rice plants grown under R/B = 4 treatment increased 88 and 53% than those grown under R treatment at the measured irradiance of 1600 and 200 μmol⋅m^−2^⋅s^−1^, respectively. Hogewoning et al. (2010b) tested the effect of different B fractions on leaf photosynthesis of cucumber seedlings. They found that *P*_n_ and *A*_max_ increased with increasing B fraction up to 50% (irradiance: 100 μmol⋅m^−2^⋅s^−1^; photoperiod: 16 h⋅d^−1^). This result was supported by analyzing the effects of different R/B ratios on Chl, stomatal characteristics, chlorophyll fluorescence, nitrogen content and carbohydrate content.

Chlorophyll is the pigment used for absorbing red and blue light, and Chl a is the molecule that makes photosynthesis possible (Johkan et al., 2010). An increase in Chl with decreasing R/B ratio was shown in **Table 1**. A higher Chl could increase light absorption, which was beneficial for *P*_n_. Earlier studies have shown that B-deficiency was adverse to chlorophyll biosynthesis in wheat seedling (Tripathy and Brown, 1995), spinach (Matsuda et al., 2008), Rosa × hybrida (Terfa et al., 2013), and cucumber seedling (Hogewoning et al., 2010b; Hernández and Kubota, 2016). Plants grown under mixture of R and B or B treatments had a higher Chl a/b compared with R treatment (**Table 1**). A higher Chl a/b indicates a high light-adapted photosynthetic apparatus with less Chl b containing light-harvesting antennae, and thereby a higher capacity for electron transport and more Calvin cycle enzymes on a Chl basis (Evans, 1988). Therefore, plants grown under mixture of R and B or B treatments had higher *P*_n_ on a Chl basis in the current study.

B is perceived directly by phototropins and activates a signaling cascade that results in fast stomatal opening under background R (Shimazaki et al., 2007). Leaves exhibited higher *g*_s_ with decreasing R/B ratio. The lowest *P*_n_ and *A*_max_ under R treatment were attributed to unresponsive *g*_s_ to increasing irradiance (**Figure 4**) and stomatal limitation. This was confirmed by the highest *L*_s_ under R treatment (**Figure 1C**). Similar result was found by Hogewoning et al. (2010b), who reported that R alone resulted in a more restricted diffusion into leaf and lower C_i_aaaC_a_^−1^ in cucumber seedlings compared with R supplemented with B. Correlation analysis between *P*_n_ and *g*_s_, *g*_m_ and *L*_s_ indicated that an increase in *P*_n_ with decreasing R/B ratio was mainly due to increased *g*_m_ and *g*_s_ and decreased stomatal limitation by decreasing R/B ratio (**Figure 2**). Although single stomatal size and stomatal pore area at the growth irradiation had a slight decrease with decreasing R/B ratio, stomatal density appeared to significantly increase (**Table 4**), resulting in an increase in *g*_s_. These results suggested that there was a direct effect of B fraction on stomatal development, further affecting photosynthesis.

R is considered as the most efficient wavelength for photosynthesis. McCree (1972) reported that the relative quantum efficiency of R (600–700 nm) was higher than that of B (400–500 nm), because fractional B was absorbed by flavonoids in vacuoles and/or pigments (anthocyanins) without function for photosynthesis in chloroplasts or less efficient in transferring energy to the reaction centers. However, it should be noted that plants grown under R treatment had the lowest *P*_n_ and *A*_max_ from all the treatments (**Figure 1A** and **Table 2**). A lower α under R treatment indicated that there were problems in photosystem. Similar result was also reported by Hogewoning et al. (2010b), who found that in cucumber seedlings B-deficiency led to leaf photosynthetic machinery dysfunction, resulting in lower *P*_n_ and *A*_max_. Miao et al. (2016) also reported that R alone could induce suboptimal activity of photosystems and inhibit electron transport from PSII donor side to PSI. Plants grown under B treatment also had a slight decrease in *P*_n_ in comparison with plants grown under R/B = 1 treatment. For B-grown leaves, higher *g*_s_ and *C*_i_ could not be limiting factors for slight decrease in *P*_n_. The possible explanation was imbalance of energy allocation between two photosystems (Tennessen et al., 1994). This was verified by lower *F*_v_′/*F*_m_′ and ΦPSII in plants subjected to monochromatic B (**Table 3**).

NUE increased with decreasing R/B ratio until 1(**Figure 5**), indicating that N availability didn’t limit the exertion of photosynthetic capacity (Hogewoning et al., 2010b). However, accumulation of carbohydrate in leaves should be an impact factor for *P*_n_. Higher starch content, along with a lower *P*_n_ under R treatment was shown than other treatments (**Table 5**). This result was similar with previous studies showed that the increase of sucrose and starch content under R treatment resulting from restriction of export of photosynthetic products out of the leaves (Sæbø et al., 1995; Li et al., 2013), which was not conducive to photosynthesis (Bondada and Syvertsen, 2003). Down-regulated *P*_n_ by carbohydrate accumulation in source leaves was a response to limited sink demand (Franck et al., 2006).

### Conflicting Effects on Growth and *P*_n_ Under Different R/B Ratios

As shown in **Table 1** and **Figure 1A**, an increase in shoot dry weight, LA and leaf number with increasing R/B ratio was observed, while opposite trend was shown in *P*_n_. Similar results has been reported in previous studies in cucumber seedlings (Hernández and Kubota, 2014, 2016). Hernández and Kubota (2016) found that shoot dry weight decreased with increasing B fraction ranging from 10 to 75%, along with decreased LA and increased *P*_n_. Hernández and Kubota (2014) reported that cucumber seedlings showed a reduction in shoot dry weight, leaf number, and LA with an increase of B fraction and no significant difference for *P*_n_ in a greenhouse with supplemental LED lighting (5.2 ± 1.2 mmol⋅m^−2^⋅s^−1^). One of the possible explanations for this result was that shoot dry weight accumulation of lettuce plant was determined not only by *P*_n_, but also other related factors, such as LA, leaf number. Furthermore, *P*_n_ measured for single leaf cannot represent *P*_n_ of entire canopy and/or whole plant (Yorio et al., 2001). Instead, variation in LA was much more efficient in determinant of variation for plant growth rate than variation in *P*_n_ before the canopy achieves full light interception (Gifford and Jenkins, 1981). Hence, compared with R/B = 12 treatment, although *P*_n_ under B, R/B = 1, R/B = 4, and R/B = 8 treatments was 12–32% higher, 6–43% (except for higher leaf number under R/B = 8 treatment) reduction in LA and 11–29% decline in leaf number under B, R/B = 1, R/B = 4, and R/B = 8 treatments resulted in decreasing shoot dry weight accumulation with decreasing R/B ratio. Another possible explanation for increasing shoot dry weight with increasing R/B ratio was due to the fact that plant exhibited greater puffiness with loose plant structure, induced by elongation of stem and leaf petiole with increasing R, leading to much more photosynthetic active radiation to be captured for growth.

Decreasing LA with decreasing R/B ratio was similar to leaf response to high irradiance (Poorter et al., 2009; Hogewoning et al., 2010b). Decreased R/B ratio might provoke high irradiance response of decreasing LA (Hernández and Kubota, 2014), which was associated with restriction of cell expansion or division induced by B (Appelgren, 1991; Folta et al., 2003; Dougher and Bugbee, 2004). Leaf extension in the vertical and horizontal directions is controlled by different genes. Blue light causes an imbalance in expression of these genes, resulting in inhibition of leaf expansion (Tsukaya, 1998). However, the effect of B dose on LA was species and cultivars specific. For example, Dougher and Bugbee (2001) tested the effect of B fraction on LA under high pressure sodium (HPS) and metal halide lamps (MH), creating five B fractions. They found LA of lettuce increased with increasing B fraction from 0 to 6% at 200 μmol⋅m^−2^⋅s^−1^ and from 0 to 2% at 500 μmol⋅m^−2^⋅s^−1^ under HPS treatments; yet there was little response to different B fraction under MH treatments (6, 12, and 26%, B fraction). In this study, LA under R and R/B = 12 treatments had no significant difference. The differences in these results suggested that other wavelengths except R and B in HPS or MH have promoting or inhibiting effects on leaf extension. In addition, increasing leaf number with increasing R/B ratio up to 12 was due to shorter growth stage induced by increasing R (Ohashi-Kaneko et al., 2007).

Although *P*_n_ under R treatment was significantly lower than those under R/B = 12 treatment, no significant difference was found in shoot dry weights under R and R/B = 12 treatments, which were higher than other mixture of R and B treatments or B treatment. Similar results were also found in tomato, salvia, and petunia (Wollaeger and Runkle, 2014), and in lettuce, tomato, and komatsuna (Ohashi-Kaneko et al., 2007). For example, Wollaeger and Runkle (2014) reported that shoot dry weight of tomato, salvia, and petunia under R treatment increased by 48–112% compared to those under R supplemented with 25% or greater B treatments, along with increasing 47–130% greater LA. Ohashi-Kaneko et al. (2007) also reported that shoot dry weight of lettuce, tomato, and komatsuna under R treatment were 14–29, 10–16, and 44–52% higher than those under mixture of R and B treatments and B treatment, respectively. In this study, this result was partly because R promoted petiole and stem elongation (Kim et al., 2004), resulting in loose leaf structure to capture much more light for growth. On the other hand, plants grown under R treatment had no reduction in LMA, LA and leaf number compared to plants grown R/B = 12 treatment, not affecting light interception. Similarly, for the above parameters, there were no significant differences between R/B = 12 and R/B = 8 treatments. However, compared with R/B = 12 treatment, a slight reduction in dry weight under R/B = 8 treatment was due to higher *R*_d_ (**Table 2**). Based on above results, there might be a maximum threshold value of B for optimal lettuce growth under R-based light source.

## Conclusion

In this study, our results showed that compared with monochromic R or B, a combination of R and B was much more efficient in facilitating lettuce growth and photosynthesis. Lettuce plants under R/B = 1 treatment exhibited the highest *P*_n_ and *A*_max_. An increase of *P*_n_ and *A*_max_ with decreasing R/B ratio was mainly associated with stomatal characteristics. However, the highest shoot dry weight was observed under R/B = 12 treatment with the greatest leaf number and LA. There was no positive relationship between *P*_n_ of single leaf and shoot dry weight accumulation. Therefore further studies should be constructed to investigate the relationship between *P*_n_ of entire plant and dry weight accumulation.

## Author Contributions

JW carried out the measurements, data analysis and drafted the manuscript. WL participated in part of measurements and data analysis. YT and QY made substantial guide about experiment design, and critically revised the manuscript.

## Conflict of Interest Statement

The authors declare that the research was conducted in the absence of any commercial or financial relationships that could be construed as a potential conflict of interest.

## Abbreviations

*A*_max_: photosynthetic capacity
Ab: abaxial surfaces of lettuce
Ad: adaxial surfaces of lettuce
B: blue light
*C*_i_: intercellular carbon dioxide concentration
Chl: chlorophyll content
Chl/LA: chlorophyll content per leaf area
*F*_s_: the light-adapted steady state fluorescence
FW: fresh weight
*F*_v_′/*F*_m_′: the efficiency of excitation capture by open PSII center
*g*_m_: apparent mesophyll conductance
*g*_s_: stomatal conductance
*L*_s_: stomatal limitation value
LA: leaf area
LED: light-emitting diode
LMA: leaf mass area
N: nitrogen content per unit dry weight
N/LA: nitrogen content per unit LA
NUE: *A*_max_ per unit N/LA
*P*_n_: photosynthetic rate
R: red light
R/B ratio: the ratio of R to B
VPD: vapor pressure difference
α: photochemical efficiency at low light
ΦPSII: the effective quantum yield of PSII.
